# Therapy-Related Myeloid Neoplasm: Biology and Mechanistic Aspects of Malignant Progression

**DOI:** 10.3390/biomedicines12051054

**Published:** 2024-05-10

**Authors:** Serena Travaglini, Massimiliano Marinoni, Valeria Visconte, Luca Guarnera

**Affiliations:** 1 Department of Experimental Medicine, University of Rome Tor Vergata, 00133 Rome, Italy; 2 Department of Biomedicine and Prevention, University of Rome Tor Vergata, 00133 Rome, Italy; 3 Department of Translational Hematology & Oncology Research, Taussig Cancer Institute, Cleveland Clinic, Cleveland, OH 44195, USA

**Keywords:** Therapy-related Myeloid Neoplasm, t-MN, Myeloid Neoplasm post cytotoxic therapy, MN-pCT

## Abstract

Therapy-related myeloid neoplasms (t-MN) arise after a documented history of chemo/radiotherapy as treatment for an unrelated condition and account for 10–20% of myelodysplastic syndromes and acute myeloid leukemia. T-MN are characterized by a specific genetic signature, aggressive features and dismal prognosis. The nomenclature and the subsets of these conditions have changed frequently over time, and despite the fact that, in the last classification, they lost their autonomous entity status and became disease qualifiers, the recognition of this feature remains of major importance. Furthermore, in recent years, extensive studies focusing on clonal hematopoiesis and germline variants shed light on the mechanisms of positive pressure underpinning the rise of driver gene mutations in t-MN. In this manuscript, we aim to review the evolution of defining criteria and characteristics of t-MN from a clinical and biological perspective, the advances in mechanistic aspects of malignant progression and the challenges in prevention and management.

## 1. Introduction

Therapy-related myeloid neoplasms (t-MN) are a subgroup of myeloid malignancies, including myelodysplastic syndromes (MDS), MDS/myeloproliferative neoplasms (MDS/MPN) and acute myeloid leukemia (AML) arising from non-correlated conditions treated with cytotoxic therapies. Advances over the years led to an increase in diagnoses and better response rates in solid tumors and autoimmune conditions, making long-term complications, such as t-MN, an increasing issue [1,2].

t-MN accounts for 10–20% of all MDS/AML, and the risk of onset in patients undergoing cytotoxic therapy ranges from 1 to 10%, depending on the type of cancer and treatment, representing one of the worst long-term side effects [3,4,5]. Particularly, primary tumors, lymphoid malignancies and gynecologic cancers are the most common types of tumors, with a 4.7-fold increased risk compared to the general population, as reported by Morton et al. [4].

t-MN, in fact, arises more commonly in older patients, carrying multiple comorbidities and with lower fitness, and unlikely to undergo intensive treatments [6].

Furthermore, the inherent features of the disease, characterized by high-risk cytogenetics and molecular alterations, make the management of t-MN a challenge for the physician [7,8,9].

Thus, both disease-related and patient-related features explain the lower response rate of t-MN, when compared to de novo MN, and the poor prognosis, standing at <10% at 5 years [6].

In recent years, a better understanding of the processes of leukemogenesis and advances in molecular biology allowed us to identify new potential factors predisposing to the development of t-MN while increasing the knowledge of the genomic landscape of these malignancies. Consequently, the classification of these neoplasms evolved over time, as well as the treatment, with a perspective of management tailored to molecular features of disease and patient characteristics.

## 2. Pathophysiology

**Figure 1:**
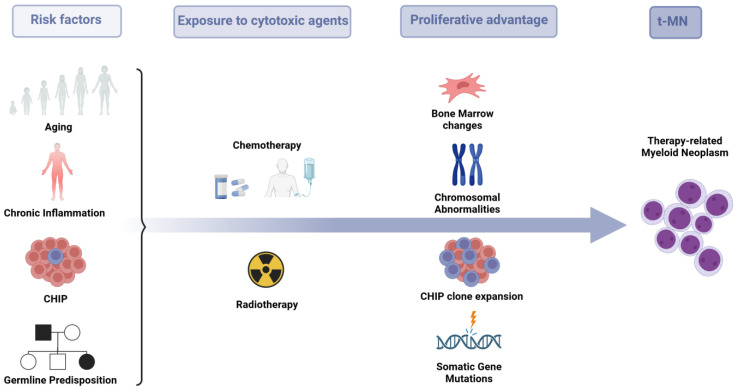
Pathophysiology of therapy-related myeloid neoplasm. The onset of t-MN is a complex, multi-step process in which, also due to germline or acquired predisposing factors, a malignant clone acquires a proliferative advantage in a microenvironment where bone marrow changes and/or genetic lesions occur.

Accumulating evidence indicates that the development of t-MN is the consequence of a complex interplay of factors, including aging, inflammation, inherited genetic susceptibility and clonal selection of a pre-existing clone that exhibits resistance to treatment and allows for genetic instability (Figure 1).

These groundbreaking advances not only shed light on the pathophysiology of therapy-related malignancies but are also paving the way for new disease classifications and clinical management of both primary malignancies and t-MN [10,11].

### 2.1. Germline Predisposition

The recognition of a germline predisposition to the development of MN was prompted by the observation of a prior history of cancer in the majority of t-MN patients [12,13,14], as well as the occurrence of MN in patients who had previously experienced an independent neoplasm and had not undergone chemotherapy or radiotherapy treatment [15,16].

Over the past decade, the development of cutting-edge technologies, including Next Generation Sequencing (NGS), has allowed to pinpoint genetic germline variants in 16–21% of t-MN patients, including inherited mutations in certain cancer-related genes, such as *BARD1*, *BRCA1*, *BRCA2*, *CHEK2*, *TP53*, as well as variants in the Fanconi Anemia pathway (*FANCA*, *FANCD2*, *FANCJ* and *PALB2*) [17,18,19,20]. Despite the fact that the clinical and biological significance of these variants has not been fully elucidated, many of these mutations affect genes involved in DNA repair pathways, cell cycle and apoptosis regulation, metabolism of genotoxic agents and various mechanisms related to cancer [5,21,22]. However, the lack of extensive and independent study cohorts, along with the absence of appropriate controls, warrants further investigations into the association between the development of secondary neoplasia and the presence of these polymorphisms [23,24,25,26].

The identification of germline variants in patients with t-MNs, coupled with the presence of a prior history of cancer in a significant proportion of cases, led to the establishment of a novel entity of myeloid neoplasms with germline predisposition in the 2016 World Health Organization (WHO) classification of hematopoietic tumors [27,28]. This category encompasses *GATA2* (GATA-binding protein 2), *CEBPA* (CCAAT enhancer binding protein alpha), *DDX41* (DEAD-Box Helicase 41), *RUNX1* (runt-related transcription factor 1), *ANKRD26* (ankyrin repeat domain containing 26) and *ETV6* (ETS Variant Transcription Factor 6) genes, which broadened the range of genetic factors contributing to inherited myeloid malignancies. It is noteworthy to highlight that the relevance of these genetic discoveries becomes even more evident when considering that nearly 60% of patients diagnosed with t-MNs have close relatives with a history of breast, ovarian or pancreatic cancer [17]. Particularly, in a retrospective analysis of t-MN patients who had a previous history of breast cancer, Churpek and colleagues revealed that roughly 20% of these patients carried mutations in known breast cancer susceptibility genes, suggesting a potential link between these conditions and a specific gene signature [17]. Similarly, it has also been observed by Schulz et al. the presence of mutations in genes linked to various familial cancers, highlighting the important contribution of genetic underpinning and familial predisposition to the individual susceptibility of t-MN [18].

There is also a higher chance of developing t-MNs in patients harboring hereditary cancer syndrome, such as Li Fraumeni syndrome, associated with the early onset of multiple neoplasms, suggesting the crucial role of *TP53* germline mutation in the development of secondary malignancies [29,30,31]. Accordingly, germline pathogenic variants in *TP53* have been documented in t-MN, even in the pediatric setting [32]. These observations strongly support the potential influence of individual predisposition, further highlighting the intricate interplay between genetic factors and the development of t-MN [17]. Moreover, the significant role of germline predisposition and familial recurrence has important clinical implications, especially when considering the selection of family donors for allogeneic stem cell transplantation, underscoring the primary importance of germline screening for MN predisposing mutations [33,34].

### 2.2. The Role of Clonal Hematopoiesis

In addition to the impact of germline variants, a crucial factor in the development of t-MN is the emergence and expansion of pre-existing hematopoietic stem cell (HSC) clones. These small populations of cells are defined by stochastically acquired mutations that improve their proliferative and survival capabilities compared to their normal counterpart [35]. Notably, the exposure to cytotoxic stress could act as a positive selective pressure, facilitating the emergence of driver gene mutations. This occurrence may arise within the context of clonal hematopoiesis of indeterminate potential (CHIP), which sets the stage for potential neoplastic transformation [36].

Several studies reported that a broad number of t-MN cases, ranging from 20% to 60%, exhibited somatic mutations in well-known genes associated with CHIP, including *DNMT3A*, *TET2*, *ASXL1*, *TP53* and *PPM1D* [37,38,39,40,41]. Particularly, the real contribution of pre-existing clones harboring somatic mutations to the susceptibility of t-MN development has been thoroughly elucidated in two case-control studies. In the study of Gillis et al., the authors explored the prevalence of CHIP in a cohort of elderly patients (≥70) with a previous history of cancer, specifically comparing patients who subsequently developed t-MNs to those who did not. Patients with CHIP displayed a higher risk of developing t-MNs. Additionally, the distribution of CHIP-related mutations varied between the two groups, with the highest prevalence observed in the group of patients with t-MN (62%) compared to the control group (27%) (*p* = 0.024) [38]. Comparable findings were achieved by Takahashi and colleagues through the examination of 14 cases, encompassing various types of cancers, and 54 lymphoma cases, serving as controls. CHIP was detected in 71% of the 14 cases, whereas only 31% of controls exhibited CH (*p* = 0.0008). *RUNX1*, *TP53*, *SRSF2* and *TET2* were the most frequently mutated genes linked to CHIP in patients who subsequently developed t-MN. Additionally, patients with CHIP had a significantly higher incidence of t-MN at 5 years follow-up compared to those without, underlying the potential significance of detecting CH as a mean to identify cancer patients at risk for developing t-MN [39].

To assess the occurrence and clinical implication of CHIP, Coombs and colleagues analyzed paired tumor and blood samples from 8810 individuals with non-hematologic malignancies, using in-depth coverage and targeted NGS data. Their findings displayed that approximately 25% of patients at diagnosis exhibited CHIP, with a significant positive correlation with several factors, including older age, prior radiation exposure and cigarette smoking, and a negative relationship with the overall survival (OS) of these patients. Strikingly, the occurrence of somatic mutations in DNA damage repair (DDR) genes such as *CHEK2*, *PPMD1* and *TP53* genes has been statistically linked to prior exposure to both chemotherapy and radiotherapy, increasing the susceptibility to subsequently develop t-MN [42].

Furthermore, Bolton et al. analyzed a large repository of specimens before and after receiving chemo-radiotherapy and detected, in most pre-therapy samples, the presence of CH. In particular, among all the somatic mutations, the ones in DDR genes showed not only higher clonal growth after treatment, but CH clones with DDR mutations grew faster compared with clones with other CH mutations in the same patient, demonstrating, thus, that cancer therapy selects the clones harboring these mutations [10].

In this perspective, Sperling et al., prompted by the observation of the association between thalidomide analogs exposure and *TP53*-mutated t-MN onset, showed, in mouse models, a survival advantage in *TP53*-mutated HSC clones when exposed to Lenalidomide, although without conferring resistance to Pomalidomide. Further analyses showed that this mechanism was mediated by differential degradation of CK1α, less targeted by Pomalidomide. Thus, upon exposure to Lenalidomide, the enhanced degradation of CK1α, which physiologically binds MDM2, mediating ubiquitination and degradation of p53, would bring about survival advantage in *TP53*-mutated clones [43,44].

Recent research has also revealed a concerning association between CHIP and the emergence of t-MN in patients with Chronic Lymphocytic Leukemia (CLL) following chemo-(immuno)therapy treatment. In one study, 30 pathogenic or likely pathogenic variants were identified in 77% of patients who later developed t-MN, compared to 12% of patients in the control group who received the same treatment. Remarkably, upon retrospective analysis, the same variants were identified in 62.5% of patients at the time of CLL diagnosis, suggesting their pre-existing and clonal nature [45].

Furthermore, a study by Awada et al. suggests that patients with CHIP-derived post-autologous stem cell transplantation (ASCT) t-MN could follow a more aggressive course with adverse-risk genetic features and significantly shorter latency duration following the procedure [46].

Taken together, these observations, along with the evidence indicating the presence of age-related CHIP in healthy subjects [47,48], imply the ongoing emergence of selected mutant clones exhibiting enhanced fitness within HSCs throughout life. While evidence suggests that CHIP alone might not be sufficient to trigger cancer development, a broad spectrum of additional factors, such as external and environmental exposures, therapeutic history, and immune function, warrant further investigation and may play a crucial role in the selection and expansion of specific clones. This investigation holds particular importance, given the substantial clinical implications for devising less toxic and targeted therapies in CHIP patients, especially in elderly patients, highlighting the crucial need for screening CH before initiating cytotoxic regimens. Furthermore, the evidence of a differential progression pattern of CHIP clones in healthy individuals [e.g., *DNMT3A*-driven CHIP does not show an increase in variant allele frequency (VAF) over time, contrary to the increase observed for *ASXL1*, *TET2*, *JAK2* and *TP53* mutations] [49] and the different propensity to acquisition new somatic mutations (higher in *TET2* and lower in *DNMT3A*) [49,50], alongside with different frequency of these mutations in t-MN patients (see chapter 3), suggest the need for a tailored management of the primary malignancy not only based on the presence of CHIP but also tailored on the specific somatic mutation driving CH.

Of note is that the above-mentioned seminal paper by Bolton et al. showed that by analyzing samples from patients receiving cytotoxic therapy and non-treated ones, a higher incidence of de novo mutations occurred in the treatment arm. This indicates that therapy can also have a mutagenic effect on HSCs and, thus, reconciles the CHIP progression t-MN pathophysiology with the classical theory dwelling on the direct DNA damage by chemo-radiotherapy [10,51,52].

### 2.3. Bone Marrow Microenvironment Changes

The bone marrow (BM) microenvironment, meant as the complex interactions between cells and soluble factors supporting hematopoiesis [53], has been recognized as one of the main characters in the process of leukemogenesis [54]. Leukemic stem cells not only modify the structure of BM by secreting vascular endothelial growth factor, which causes the sprouting of blood vessels, but they also stimulate granulocyte and monocyte colony-stimulating factor (GM-CSF) by endothelial cells. Furthermore, they are protected from chemotherapy in osteoblast-rich areas of the BM [54,55]. In this environment, leukemic cells undergo senescence, a complex process characterized by cell cycle arrest, which is triggered by several heterogeneous stimuli such as telomere shortening after a certain number of cell divisions, oncogene upregulation and exposure to cytotoxic treatments, reactive oxygen species (ROS) and ionizing radiations [56,57,58]. This process, physiologically, plays an important role in the protection from replication of pre-cancerous cells [53].

Leukemic cells exhibit a prolonged senescent phenotype, which results in both higher expression of p53 and p21 and secretion of several mediators, such as chemokines, cytokines, grow factors and proteases, which cause inflammation and shape a leukemic permitting environment [59,60].

Intriguingly, not only these senescent features are more pronounced in t-MN when compared to de novo MN, but, as shown by Kutyna et al., they also modify the BM niche long before the onset of the malignancy [61].

Studies on mouse models by Stoddart et al. showed that the exposure of HSCs and BM alone to alkylating agents promotes the expansion of *TP53 silenced* cells, whereas t-MN development is driven by the synergistic effects of chemotherapy exposure of premalignant hematopoietic cells, together with deleterious effects of cytotoxic therapy on the supporting microenvironment [62].

Furthermore, Özdemir et al. documented an increased expression of genes involved in xenobiotic metabolism, DNA double-strand break response, heat shock response, and cell cycle regulation in healthy BM exposed to Etoposide [63].

This evidence highlights the major role of BM niche changes in post-cytotoxic therapy leukemogenesis. In this perspective, promising data have been reported on the efficacy of senolytic agents, such as Dasatinib, Quercetin and JAK inhibitors, in selectively eliminating senescent cells, inhibiting the senescent secretome and restoring the differentiating potential of mesenchymal cells [61,64,65].

## 3. Classification of t-MN: Evolution and Novelties

**Figure 2:**
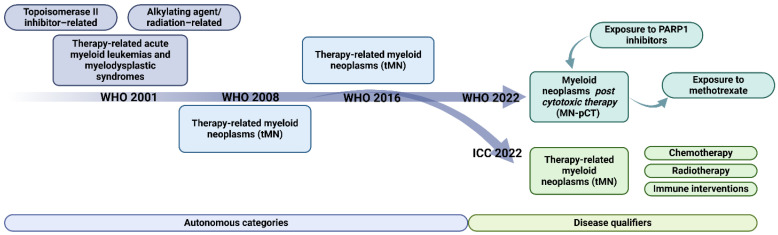
Evolution of classification of therapy-related myeloid neoplasm. Classification systems over time privileged genomic features of the disease over anamnestic information. This led to the re-scaling of the t-MN entity until reaching the status of disease qualifier. ICC: International Consensus Classification; t-MN: therapy-related myeloid neoplasia; WHO: world health organization.

The classification of secondary AML (sAML), and in particular t-AML, initially based on anamnestic and clinical criteria, has undergone profound changes over the years (Figure 2) and is currently based on the genetic-molecular characteristics of the disease.

The first distinction between de novo AML and sAML appeared in the 2001 WHO classification [66], which distinguished, in the latter leukemic subgroup, AML with multilinear dysplasia [AML-MD, whose diagnosis was established in the presence of dysplasia in at least 50% of the cells belonging to at least two myeloid cell lines on morphological examination of the bone marrow and/or a documented history of MDS or MDS/myeloproliferative syndrome (MDS/MPN), dated at least six months prior to the diagnosis of AML], and AML secondary to chemo/radiotherapy (therapy-related, in patients who had undergone prior chemo/radiotherapy for an unrelated tumor). This latter entity was further divided according to the neoplastic agent to which the patient had been previously exposed into AML arising after therapy with alkylating agents/radiotherapy, commonly arising 4–7 years after treatment, and with morphologic features suggestive of prior MDS, and AML arising after therapy with Topoisomerase II inhibitors, characterized by a shorter latency and the absence of an antecedent phase of myelodysplasia [67,68].

The 2008 classification revision [69] eliminated the latter subclassification of t-AML, which was included, along with MDS with the same features, in the group of t-MN. In addition, advances in the biology of leukemogenesis brought the recognition of a list of dysplasia-related abnormalities as a diagnostic criterion in the newborn group of AML with myelodysplasia-related changes (morphological or genetic) (AML-MRC), which replaced AML-MD [69].

In 2016, a new revision of the classification made minimal changes to the pre-existing categories (the exclusion from dysplasia-related cytogenetic abnormalities of chromosome 9 deletion, as it is often associated with *NPM1* mutations, and the biallelic *CEBPA* mutation) [27,70,71]. However, for the first time, a category of MN characterized by specific germline mutations responsible for an increased risk of developing a neoplastic clone was recognized [AML with germline predisposition (AML-GP)] [27].

Finally, two additional classifications have been recently published: the WHO 5th edition (2022) [72] and the ICC (International Consensus Classification) [73] classification system of MN, by a group of experts in the study of clinical, pathological and genetic aspects of AML.

The WHO 2022 classification excluded from secondary MN the AML secondary to a previous MPN (now included in the classification of MPN) or MDS, now defined as myelodysplasia-related AML (MR-AML). In contrast, the group of AML-GP, whose list has been significantly expanded, and AML secondary to cytotoxic therapy (AML-pCT, replacing the previous designation of therapy-related) have been confirmed and now also include patients exposed to poly(ADP-ribose) polymerase inhibitors (PARPi), while excluding those previously treated with methotrexate [72]. In contrast, all subcategories of sAML identified by previous classification systems are retained in the ICC 2022 classification: AML progressed from previous MDS, progressed from previous MDS/MPN, therapy-related, and with germline predisposition (Table 1) [73].

### PARP Inhibitors

As mentioned above, exposure to PARPi has been recently recognized as a risk for t-MN development. PARPi has been increasingly utilized in the past years as a salvage therapy for various solid cancers, including but not limited to gynecological cancers such as ovarian and breast cancer [74]. One of the earliest studies in this regard was carried out by Todisco et al. in 2020, where the recorded incidence of secondary hematologic cancers in a pool of 130 patients who have epithelial ovarian cancer (EOC) was 6.9% [75]. In a successive study from the same team, with an increased population of 182 patients, the cumulative incidence of t-MN turned out to be 8.7% [76]. Although other studies, such as the one by Almanza-Huante et al., presented a lower incidence rate of t-MN amongst EOC patients, with approximately 1% of patients exposed to PARPi developing a hematological disease, the same conclusion was reached in terms of the increased relative risk [77]. Furthermore, studies such as those from Marmouset et al. and Martin et al. highlighted how most of these patients developed t-MN characterized by an adverse risk cytogenetic and molecular profile, with over 60% of them bearing complex karyotypes and/or *TP53* mutations [78,79]. As expected, the OS of these patients is quite poor, as supported by Chiusolo et al., who reported a median OS of 5 months for their population of post-PARPi t-MN patients [80]. Finally, Morice et al. carried out a meta-analysis in 2021 across approximately 6000 patients treated with PARPi, as well as across multiple solid tumors, which confirmed the scenario: PARPi exposure after chemotherapy increases the risk of developing hematological disease (Table 2) [81].

The inclusion of PARPi into the list of potential leukemogenic agents highlights once again the importance of pharmacovigilance and awareness toward t-MN as a possible complication of solid and hematologic cancers.

## 4. Genetic Signature

**Figure 3:**
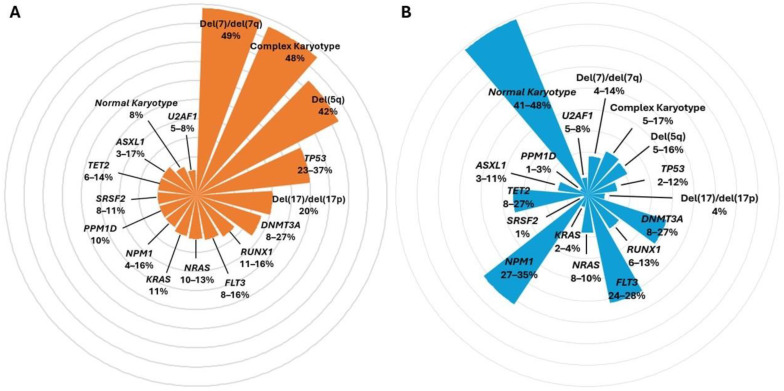
Landscape of the most common genetic features of t-MN (**A**) and de novo MN (**B**). The orange columns refer to somatic lesions and karyotype abnormalities most frequently detected in t-MN, whereas the blue ones in de-novo MN. The percentage of genetic mutations is reported from the review by McNerney et al. and Hsu et al. t-MN: therapy-related myeloid neoplasia.

There are no specific markers or cluster mutations for t-MN. Nonetheless, this class of malignancies presents a specific signature, responsible for their poor outcome (Figure 3) [82].

Analysis of karyotype reveals, when compared to de novo malignancies, a lower percentage of normal cytogenetics. In particular, anomalies frequently observed are del(5q) (20–45%), del(7)/del(7q) (30–50%), del(17)/del(17p) (5–20%) and complex karyotype (30–50%, defined as >3 karyotypic anomalies) [82,83].

Conversely, recurrent AML cytogenetic abnormalities such as t(15;17) [detected in 95–98% of acute promyelocytic leukemia (APL) [84,85] and core binding factor AML [defined by t(8;21), t(16;16), inv(16)] are rare (2% in both the entities) and the status of ‘therapy-related’ does not impact the prognosis on APL patients [82,86].

The broader availability of NGS allowed a better comprehension of the somatic mutational landscape displayed by t-MN. Several authors documented an enrichment in DDR genes, with a frequency of *TP53* mutation ranging from 15 to 40% (vs. 2–12% in de novo MN), whereas the exact incidence of *PPM1D* and *CHEK2* (genes not always included in clinical practice-NGS myeloid panels) seems to be roughly 10% (vs. 1–3% in de novo MN) and 3%, respectively [82,83,87,88].

Gene mutations commonly associated with de novo AML, such as *NPM1*, *FLT3*, *IDH1* and *IDH2*, are less observed in t-MN (4–16% vs. 27.35%, 8–16% vs. 24–28%, 3–5% vs. 8–10%, 0–5% vs. 9–10%, respectively), whereas, altogether, mutations in RAS pathway genes are more frequent: *NRAS* 10–13% vs. 8–10% in de novo MNs, *KRAS* 11% vs. 2–4%, *PTPN11* 3–9% vs. 5%, *NF1* 2–4% vs. 2%, *CBL* 2–4% vs. 1%. In a similar fashion, mutations in splicing factor genes are more common: *SETBP1* 3% in t-MN vs. 0–1% in de novo MN, *SF3B1* 0–3% vs. 1–5%, *SRSF2* 8–11% vs. 1%, *U2AF1* 5–8% vs. 4%, *ZRSR2* 1% vs. 0%. Conversely, no significant differences are observed in the incidence of DTA gene mutations: *DNMT3A* 8–27% in t-MN vs. 14–25% in de novo MN, *TET2* 6–14% vs. 8–27%, *ASXL1* 3–17% vs. 3–11% [82].

## 5. Clinical Implications and Future Perspectives

As evident from the above-showed data, t-MN is not only enriched in high-risk mutations as defined by common classification systems (ICC/WHO 2022 [72,73]: *TP53*, *U2AF1*, *SRSF2*, *STAG2*, *SF3B1*) but also present lower mutation targetable by specific therapies (e.g., *FLT3*, *IDH1*, *IDH2*).

Nowadays, the only specific drug approved for t-AML is CPX3-5-1 (VYXEOS), a dual-drug liposomal encapsulation of cytarabine and daunorubicin at a fixed 5:1 synergistic molar ratio, which showed higher efficacy when compared to standard-of-care cytarabine plus daunorubicin chemotherapy [median overall survival 12 months vs. 6 months with cytarabine and an anthracycline (7 + 3 regimen)] [89].

Since most of the patients are old and/or unfit for intensive chemotherapy, the management of t-MN remains an unmet clinical need.

Several attempts have been made to target *TP53*, one of the signature mutations of these malignancies and an independent unfavorable clinical predictor [90]. *TP53*-mutated MN presents, in fact, a lower response rate to therapy when compared to its *TP53*-wild-type counterpart, with a median OS ranging from 5 to 10 months [91].

Intensive chemotherapy in *TP53*-mutated AML presents a low rate of complete response (CR: 20–40%) with an OS of 4–9 months [92,93,94,95,96]. *TP53* mutation has also been identified as a predictor of inferior response to CPX-351 [97]. Hypomethylating agents (HMA) showed an overall response rate (ORR) of 30–100% with a CR rate of 10–20% in AML and 1–30% in MDS bearing *TP53* mutation [91,94,98]. Despite these variables’ rate of response, HMA failed to significantly improve long-term survival (OS 2–7 months in AML, 9–13 months in MDS) [98,99,100,101].

The addition of Venetoclax, a BCL-2 inhibitor, to HMA brought a higher rate of response: the seminal study by DiNardo et al. in *TP53*-mutated AML patients reported a CR/CR with incomplete hematologic recovery (CRi) of 47% [102]. These promising results were confirmed by Aldoss et al. and Kim et al., who observed a 52% and 57% CR/CRi rate, respectively [103,104]. However, again, these results did not improve the survival: none of these studies documented a median OS exceeding 7 months [102,103,104].

Considering the elderly age and low-performance status, allogeneic hematopoietic stem cell transplantation (HSCT) is rarely an option in patients with t-MN [6]. However, when feasible, it should be performed, granting better outcomes when compared to more conservative treatments [90]. Nevertheless, *TP53* mutation, especially with additional high-risk features (such as truncating mutations, high VAF and association with complex karyotype), is an independent poor prognosis predictor [105,106,107].

The unsatisfactory results of the available therapies led to the development of new strategies.

Magrolimab is a humanized IgG4 monoclonal antibody against CD47, integrin-associated protein transduction ‘don’t eat me’ signals towards macrophages and inhibiting phagocytosis [108]. CD47 is highly expressed in AML cells, enhancing tumor immune escape [109]. Thus, this surface protein has been targeted with Magrolimab in combination with both HMA and HMA + Venetoclax in *TP53*-mutated AML and MDS. The preliminary results of the combination Magrolimab-HMA showed promising results with CR/CRi rates ranging from 33 to 64% with median OS from 11 to 16 months [110,111]. The addition of Venetoclax obtained a stunning 100% CR/CRi rate in an initial evaluation of a phase Ib enrolling unfit relapsed AML patients [112]. However, despite these findings, recently FDA placed a full clinical hold on all Magrolimab AML and MDS studies after the combination of Magrolimab–Venetoclax–Azacitidine demonstrated futility and an increased risk of death in AML patients in an ENHANCE-3 study [113].

Flotetuzumab, a bispecific dual affinity re-targeting antibodies (DARTs) binding CD3 and CD123, induces cytotoxic T-cell response against cells expressing CD123, like AML blasts [114]. A post-hoc analysis of the CP-MGD006-01 clinical trial, testing Flotetuzumab in relapsed AML patients, showed a 47% CR rate in those bearing TP53 mutation; these patients had a median OS of 10 months. 

Sabatolimab, an inhibitor of TIM3, an immune regulator expressed in myeloid blasts, was tested, in combination with HMA, in a cohort of high-risk MDS/AML. In the *TP53*-mutated MN subgroup, the investigators observed an ORR of 71% with a median duration of response of 21 months [115].

Eprenetapopt (APR-246) is an agent binding the DNA in *TP53* mutant cancer cells, restoring an active wild-type-like conformation and function of p53 and showing efficacy in MN in association with HMA [116]. Cluzeau et al. documented a CR rate of 47% in MDS and 17% in AML [117]. Sallman et al., in a cohort of patients with MDS and oligoblastic AML (<30% blasts), observed a CR rate of 43% (49% MDS, 36% AML and 0% MDS/MPN) [118,119]. Of note, concordantly with the mechanism of action of the drug, isolated *TP53* mutation was predictive of higher CR rate; also, higher risk features, such as biallelic *TP53* mutation and complex karyotype, were correlated with higher CR [118,119]. Eprenetapopt-HMA combination was also tested in a post-HSCT setting for *TP53*-mutated MDS/AML as maintenance therapy, with an OS of 21 months at a median follow-up of 17 months and a 1-year OS probability was 79% [120]. However, a phase 3 clinical trial of Eprenetapopt–Azacitidine for frontline treatment of *TP53*-mutant MDS patients has been completed and failed to meet the primary statistical endpoint of CR [121]. The addition of Venetoclax to the Eprenetapopt–HMA combination in *TP53*-mutated AML showed an ORR of 64% and a CR rate of 38% (Table 3) [122].

## 6. Conclusions and Future Perspectives

The definition of t-MN has greatly evolved over time. Despite huge advances in the molecular bases of its pathogenesis, there are no specific mutations, and the assessment of t-MN still relies on the presence or absence of a history of cytotoxic therapies for unrelated disorders. In this perspective, efforts have been made to identify treatments conferring greater risk for the development of t-MN, whose paradigm is the recent identification of PARPi as a potential leukemogenic agent.

The management of t-MN and, in particular, *TP53*-mutated MN is still an unmet medical need. Nevertheless, the growing number of possible therapeutic targets [124], alongside the broader accessibility to HSCT, hold the promise of significant improvements in the near future. In particular, considering the overlapping mutational features between de novo and t-MN and the profound difference in the leukemic niche and in the staminal senescent mechanisms, the latter mechanism could be a promising target for senolytic agents which, alone or combined with chemotherapy, showed high efficacy in preclinical models [64,125].

In a similar fashion, the advances in the comprehension of the molecular pathogenesis of t-MN could bring useful strategies in order to prevent the onset of this dangerous complication. In this perspective, also thanks to the broader implementation of genetic testing, it is possible to envision the inclusion of CHIP evaluation in the prognostic and decisional algorithms of solid and hematologic cancers. This will help stratify future risks of the development of secondary malignancies, thus allowing a therapeutic strategy tailored to both patient-related risk factors and treatment-related leukemogenic potential.

## Figures and Tables

**Table 1:** t-MN definition: similarities and differences in WHO and ICC classifications. AML: acute myeloid leukemia. CCUS: clonal cytopenia of undetermined significance. MDS: myelodysplastic syndrome. MPN: myeloproliferative neoplasm. PARPi: poly(ADP-ribose) polymerase inhibitors; t-MN: therapy-related myeloid neoplasms.

	WHO5th	ICC
Denomination	Post cytotoxic therapy	Therapy-related
Entity	Disease qualifier	Disease qualifier
Condition characterized	AML, MDS, MDS/MPN (no CCUS)	AML, MDS, MDS/MPN (no CCUS)
Previous treatments	Chemotherapy (no methotrexate), radiotherapy, PARPi	Chemotherapy, radiotherapy, immune interventions
Examples	AML, myelodysplasia-related, post cytotoxic therapy	AML with myelodysplasia-related cytogenetic abnormality, therapy-related

**Table 2:** Table including the major studies of t-MN arising after PARPi treatment. AML: acute myeloid leukemia. CCUS: clonal cytopenia of undetermined significance; CK: complex karyotype; LAL: lymphoblastic acute leukemia; m: months; MDS: myelodysplastic syndromes; t-MN (therapy-related myeloid neoplasia).

Authors	Population	t-MN	MN Phenotype	Genetic Features	Primary Cancer	OS (m)	Time to t-MN (m)
Almanza-Huante et al. (2023) [77]	1462	1%	60% MDS, 34% AML, 6% MPAL		Ovarian, breast	7.8	20.7
Chiusolo et al. (2022) [80]	300	4.3%	t-AML/MDS	100% *TP53*	Ovarian cancer	5	12
Marmouset et al. (2022) [78]	373	3.5%	65% MDS, 35% AML	61% CK, 71% *TP53*	Ovarian, breast	9.6	19
Martin et al. (2021) [79]	20 (100% t-MN)		55% MDS, 45% AML	95% CK	Ovarian	4.3	24
Morice et al. (2021) [81]	5693	0.73%			Multiple		17.8
Todisco et al. (2020) [75]	130	6.9%	11% CCUS, 55% MDS, 22% AML, 11% LAL	55% del5q or del7q, 33% CK, 55% *TP53*	Ovarian		22.8
Todisco et al. (2022) [76]	182	8.7%	75% MDS, 25% AML	43% del5q or del7q, 56% CK, 56% *TP53*	Ovarian		24

**Table 3:** Approved and on-study treatments for t-MN and TP53-mutated MN. AML: acute myeloid leukemia; CHT: chemotherapy; CR: complete response; CRi: complete remission with incomplete hematologic recovery; HMA: hypomethylating agents; mDOR: median duration of response; MDS: myelodysplastic syndromes; mOS: median overall survival; MPN: myeloproliferative neoplasms; ORR: overall response rate; t-AML: therapy-related AML.

Reference	Drug	Combination	Phase	Setting	Outcome
Rücker et al., 2012 [95] Hou et al., 2015 [93] Yanada et al., 2016 [96] Stengel et al., 2017 [92]	Standard CHT	/	/	*TP53-mutated* AML	CR 20–40% mOS 4–9 months
Welch et al., 2016 [100] Short et al., 2018 [101] Boddu et al., 2018 [98] Bewersdorf et al., 2020 [99]	HMA	/	/	*TP53*-mutated MDS/AML	CR 10–20% (AML) CR 1–30% (MDS) mOS 2–7 months (AML) mOS 9–13 months (MDS)
Aldoss et al., 2019 [104] Kim et al., 2021 [103] DiNardo et al., 2019 [102]	HMA	+Venetoclax	/	*TP53*-mutated AML	ORR 47–57% mOS 5–7 months
Lancet et al., 2018 [89]	CPX3-5-1	/	III	t-AML	CR/CRi 47.7% CR 37.3% mOS 12 months
Daver et al., 2022 [111]	Magrolimab (antiCD47)	+Azacitidine	Ib	*TP53*-mutated AML	CR/CRi 48.6% CR 33.3% mOS 10.8 months
Sallman et al., 2022 [110]	Magrolimab	+Azacitidine	Ib	*TP53*-mutated high-risk MDS	CR 40% mOS 16.3 months
Daver et al., 2021 [112]	Magrolimab	+Venetoclax +Azacitidine	Ib	*TP53*-mutated unfit AML and R/R AML	CR/CRi 100%
/	Magrolimab	+Venetoclax +Azacitidine	III	Unfit ND-AML	Stopped for futility
Vadakekolathu et al., 2020 [123]	Flotetuzumab (antiCD123)	/	I/II	*TP53*-mutated R/R AML	CR 47%
Brunner et al., 2021 [115]	Sabatolimab (antiTIM3)	+HMA	Ib	*TP53*-mutated high-risk MDS	ORR 71% mDOR 21.5 months
Cluzeau et al., 2021 [117]	Eprenetapopt (p53 reactivator)	+Azacitidine	II	*TP53*-mutated high risk MDS/AML	ORR 62% (MDS) ORR 33% (AML) CR 47% (MDS) CR 27% (AML)
Sallman et al., 2021 [119]	Eprenetapopt	+Azacitidine	II	*TP53*-mutated MDS/oligoblastic AML	CR 49% (MDS) CR 36% (AML) CR 0% (MDS/MPN)
Mishra et al., 2022 [120]	Eprenetapopt	+Azacitidine	II	After BMT in *TP53*-mutated MDS/AML	mRFS 12.5 months mOS 20.6 months
Garcia-Manero et al., 2023 [122]	Eprenetapopt	+Venetoclax +Azacitidine	I	*TP53*-mutated AML	CR/CRi 64% CR 38%
/	Eprenetapopt	+Azacitidine	III	*TP53*-mutated MDS	Stopped for futility

## Abbreviations

ANKRD26	ankyrin repeat domain containing 26
AML	acute myeloid leukemia
APL	acute promyelocytic leukemia
ASCT	Autologous stem cell transplantation
BM	bone marrow
CCUS	clonal cytopenia of undetermined significance
CEBPA	CCAAT enhancer binding protein alpha
CK	complex karyotype
CLL	chronic lymphocytic leukemia
CR	complete remission
CRi	complete remission with incomplete hematologic recover
CHIP	clonal hematopoiesis of indeterminate potential
CHT	chemotherapy
DART	dual affinity re-targeting antibodies
DDR	DNA damage repair
DDX41	DEAD-Box Helicase 41
DOR	Duration of response
EOC	epithelial ovarian cancer
ETV6	ETS Variant Transcription Factor 6
GATA2	GATA-binding protein 2
GM-CSF	granulocyte and monocyte colony-stimulating factor
GP	germline predisposition
HMA	hypomethylating agents
HSC	hematopoietic stem cell
HSCT	hematopoietic stem cell transplant
ICC	international consensus classification
LAL	lymphoblastic acute leukemia
M	months
MDS	myelodysplastic syndromes
MPN	myeloproliferative neoplasms
MRC	myelodysplasia-related changes
NGS	next generation sequencing
ORR	overall response rate
OS	overall survival
PARPi	poly(ADP-ribose) polymerase inhibitors
pCT	post-cytotoxic therapy
ROS	reactive oxygen species
RUNX1	runt-related transcription factor 1
t-MN	therapy-related Myeloid Neoplasms
VAF	variant allele frequency
WHO	world health organization
